# Clinical and genetic characteristics of cystic fibrosis in CHINESE patients: a systemic review of reported cases

**DOI:** 10.1186/s13023-018-0968-2

**Published:** 2018-12-17

**Authors:** Xiaobei Guo, Keqiang Liu, Yaping Liu, Yusen Situ, Xinlun Tian, Kai-Feng Xu, Xue Zhang

**Affiliations:** 1 Department of Respiratory and Critical Care Medicine, Peking Union Medical College Hospital, Chinese Academy of Medical Sciences & Peking Union Medical College, #1 Shuaifuyuan, Wangfujing, Beijing, 100730 China; 2 Emergency Center, Beijing Tongren Hospital, Capital Medical University, Beijing, China; 3 McKusick-Zhang Center for Genetic Medicine, State Key Laboratory of Medical Molecular Biology, Institute of Basic Medical Sciences, Chinese Academy of Medical Sciences & Peking Union Medical College, Beijing, 100005 China; 4 Department of Biochemistry, Faculty of Medicine and Dentistry, University of Alberta, Edmonton, Canada

**Keywords:** *CFTR*, Chinese patients, Cystic fibrosis, Genetics, Phenotype

## Abstract

Cystic fibrosis (CF) is a rare disease most commonly seen in Caucasians. Only a few Chinese CF patients have been described in literature, taking into account the large population of China. In this systematic review, we collected the clinical and genetic information of 71 Chinese CF patients based on all available data. Compared with Caucasians, Chinese CF patients often present atypical symptoms, mainly displaying symptoms of pulmonary infection with fewer digestive symptoms. An ethnicity-specific *CFTR* variant spectrum was also observed in CF patients of Chinese origin, with p.Gly970Asp as the most common mutation while p.Phe508del, the most common pathogenic mutation in CF patients of Caucasian origin, is rare, suggesting the necessity of a Chinese-specific *CFTR* variant screening panel. Besides, multiplex ligation-dependent probe amplification analysis should be routinely considered, especially for those with unidentified mutations. Potential under-diagnosis of CF in Chinese patients might be caused by a combination of atypical clinical features and genetic heterogeneity in Chinese CF patients, the inaccessibility of sweat and genetic testing facilities, and the one-child policy in China. With the approval of promising small molecule correctors and potentiators, molecular characterization of Chinese-specific *CFTR* mutations will help to realize more precise treatment for Chinese CF patients.

## Background

Cystic fibrosis (CF, OMIM # 219700) is considered to be a rare autosomal recessive disease involving multiple organs, especially the lungs and digestive organs. CF is most commonly seen in the Western world, affecting about 1/4000 newborns in the US and having higher morbidity in some European countries. [1] It is a multisystem disease caused by mutations in the cystic fibrosis transmembrane conductance regulator *(CFTR)* gene located in chromosome 7, which encodes the chloride ion channel. [2, 3] Since the *CFTR* gene was finally isolated as the pathogenic gene of CF in 1989, [2, 4, 5] over 2000 mutations have been identified in CF patients according to the Cystic Fibrosis Mutation Database (http://www.genet.sickkids.on.ca). The variant spectrum of *CFTR* among Caucasians in Western countries has been very well established.

Compared with the relatively higher morbidity in Caucasians, however, much fewer CF patients have been reported in Asia, and the incidence varies greatly from 1:10000 to 1:40750 among different countries. [6, 7] Up to now, only approximately 70 CF patients of Chinese origin were reported in literature. Yang and colleagues enrolled 229 Chinese individuals with chronic diarrhea or chronic upper or lower respiratory infection, which are symptoms that are similar to CF, but none of the patients were detected with abnormally elevated sweat Na^+^. Therefore, they concluded that CF might be quite rare in China. [8] Interestingly, more than 50 CF patients of Chinese origin have been diagnosed in the last ten years, two and half times more than the number of diagnoses in the previous 30 years (1975–2006) since the first case was reported in 1975, [9] which suggests that there might have been more unrecognized CF patients in the past. Unpublished data provided by WuXi NextCODE Ltd. showed that the carrier frequency of CF in China is about 1/127, using data from 3058 healthy adults in their Chinese database, and that the estimated CF incidence in China should be around 1/64,000. Even if CF remains a rare disease, considering the large population in China, the expected number of CF patients might be over 20,000 based on a population of 1.39 billion. Thus, potential CF patients must be screened more effectively and precisely. However, due to inadequate awareness of the disease by Chinese physicians, atypical manifestations and a different mutation spectrum compared to Caucasians, combined with the inaccessibility of sweat and genetic testing facilities, the diagnosis of CF still remains very difficult in Chinese.

In this paper, we summarize the clinical and genetic characteristics of Chinese CF patients, in the hope of improving the awareness of CF by Chinese physicians and suggesting a direction of target therapy.

## Methods

### Information sources and search strategies

Studies were identified by searching Pubmed, Embase, Cochrane Library, OVID medicine and SinoMed from January 1, 1975 to May 31, 2018. The search strategy included the following term keys: (‘cystic fibrosis’) AND (‘Chinese’ OR ‘China’). Publications were restricted in HUMAN written in English or Chinese during the literature searching. Study types were limited to clinical trial, meta-analysis, randomized controlled trial, case report, case series or review. In addition, we reviewed the references of included articles as a complement of related articles not included in the initial search. We also contacted authors of relevant papers regarding further published and unpublished work.

### Study selection

Two reviewers (X. G. and K. L.) independently performed the initial search and the eligibility assessment. X. G. and K. L. respectively screened out the related studies through titles and abstracts of all articles. Disagreements were resolved by consensus between all authors. Then, full text articles were assessed for eligibility by the authors.

### Inclusion and exclusion criteria

Original articles were included if they met the inclusion criteria: 1) Diagnosis in accordance with cystic fibrosis; 2) patients were of Chinese origin; 3) clinical or genetic data could be found in the full text.

Exclusion criteria were as follows: 1) duplicate publications; 2) interim or extension findings of the same study, or with duplicate patients; 3) studies without detailed clinical and genetic data.

### Data collection

Data was extracted from all eligible articles using standardized excel forms. Data retrieved from the articles included: 1) basic information of studies: first author name, publication year, study location, study design, number of participants and treatment; 2) methodological qualities of the study; 3) clinical symptoms including: a) basic information of patients: gender, age at onset of symptoms, age at diagnosis, height and weight at diagnosis, family history; b) respiratory symptoms: sinus disease or nasal polyps, acute or persistent respiratory abnormalities, pulmonary function including forced expiratory volume in one second (FEV_1_) percent predicted, sputum culture and allergic bronchopulmonary aspergillosis (ABPA); c) digestive tract symptoms: meconium ileus or other intestinal obstruction, failure to thrive or malnutrition, liver problems, steatorrhea/malabsorption; d) congenital bilateral absence of vas deferens (CBAVD) or infertility; 4) diagnostic tests information: Cl^−^ concentration in sweat chloride testing and details about genetic screening of *CFTR* including nucleotide change, amino acid change and screening method.

### Diagnosis of cystic fibrosis

Cystic fibrosis was diagnosed if patients met the inclusion criteria according to the 2017 consensus guidelines for CF diagnosis from the Cystic Fibrosis Foundation. [1] More concretely, the full text of all studies was reviewed, and if the diagnosis of CF in a specific study can be made following the 2017 consensus guidelines mentioned above, then the study would be included in this systematic review. According to the guidelines, CF diagnosis will be made if a patient has CF clinical features or a positive family history with one of the following: 1) Sweat chloride value is ≥60 mmol/L; 2) Sweat chloride values in the intermediate range (30–59 mmol/L) and with 2 CF-causing *CFTR* mutations or CFTR dysfunction approved by CFTR physiologic testing; 3) Individuals with clinical features that may be consistent with CF who have a sweat chloride< 30 mmol/L indicates that CF is less likely.

### Nutritional status assessment

Nutrition outcomes were evaluated through weight and height. Patients were divided into two age groups to report nutrition metrics: infants and preschoolers younger than 7 years old; school-age children and adolescents older than 7 years. Goals for nutrition metrics for the older group were based on the Comprehensive Evaluation of Children and Adolescent Development (2014 version, available at http://www.nhfpc.gov.cn/zhuz/pqt/201504/3661756c241b46329dbc6ad73eba0bd1.shtml), while those for the younger group were based on the Growth and Development References Standard of Chinese Children Younger than 7 Years of Age (2009 version, available at http://www.nhfpc.gov.cn/fys/s7906/200910/994a7f6e1bd1491a9e8efa8e762a313f.shtml) which has some differences from the WHO growth curves.

### Pancreatic insufficiency

The most commonly used test to screen for/diagnose PI in individuals with CF is the fecal pancreatic elastase−1 assay. When a value of < 100 μg/g is used, the specificity and sensitivity of fecal pancreatic elastase-1 in a pediatric CF cohort is 100%. [10] In China, most clinical centers were unable to test fecal pancreatic elastase levels; instead, fecal fat microscopy by Sudan III was usually used to assess pancreatic function. Results were reported as negative or positive. Patients with steatorrhea and positive results on these tests were considered to have pancreatic insufficiency to some extent.

### Congenital bilateral absence of vas deference (CBAVD)

Diagnosis of CBAVD was achieved based on these criteria: [11] the presence of normal to slightly smaller testicles, non-palpable vas deferens, normal plasma levels of FSH (follicle-stimulating hormone), and reduced ejaculate volume (< 1 mL).

### Diagnosis criteria of allergic bronchopulmonary aspergillosis (ABPA)

Diagnosis of ABPA was achieved according to the consensus criteria of the cystic fibrosis foundation in 2003 [12]: 1) an unexplained acute or subacute pulmonary function exacerbation; 2) a total serum Immunoglobulin E concentration greater than 1000 IU/mL, unless the patient was receiving corticosteroids; 3) an immediate cutaneous reaction to *Aspergillus fumigatus* or serum IgE antibody to *A. fumigatus*; 4) precipitating antibodies to *A. fumigatus* or serum IgG antibody to *A. fumigatus* by an in vitro test; 5) New or recent abnormalities found via chest radiography (infiltrates or mucus plugging) or chest CT (bronchiectasis) that have not cleared with antibiotics and standard physiotherapy.

### Mutation nomenclature

Mutation information obtained from original publications was reconciled according to the HGVS-nomenclature, which is recommended as an international standard for the description of sequence variants. [13] Mutations were described at both DNA and protein levels. The variant positions were recorded according to the reference coding DNA sequence (NM_00492.3). Mutations with legacy names were checked manually and translated into the form recommended by HGVS-nomenclature.

### Data analysis

Data analysis was conducted using SPSS version 22.0 software (IBM SPSS, USA). All continuous variables were evaluated for a normal distribution with a Kolmogorov–Smirnov test. Parametric data is presented as the mean ± standard deviation, and non-parametric data is presented as the median and extreme values. Categorical variables are presented as either a percentage of the total or as absolute values. Variant frequency and percentages were calculated using data on patients with definite genotype information.

## Results

**Fig. 1:**
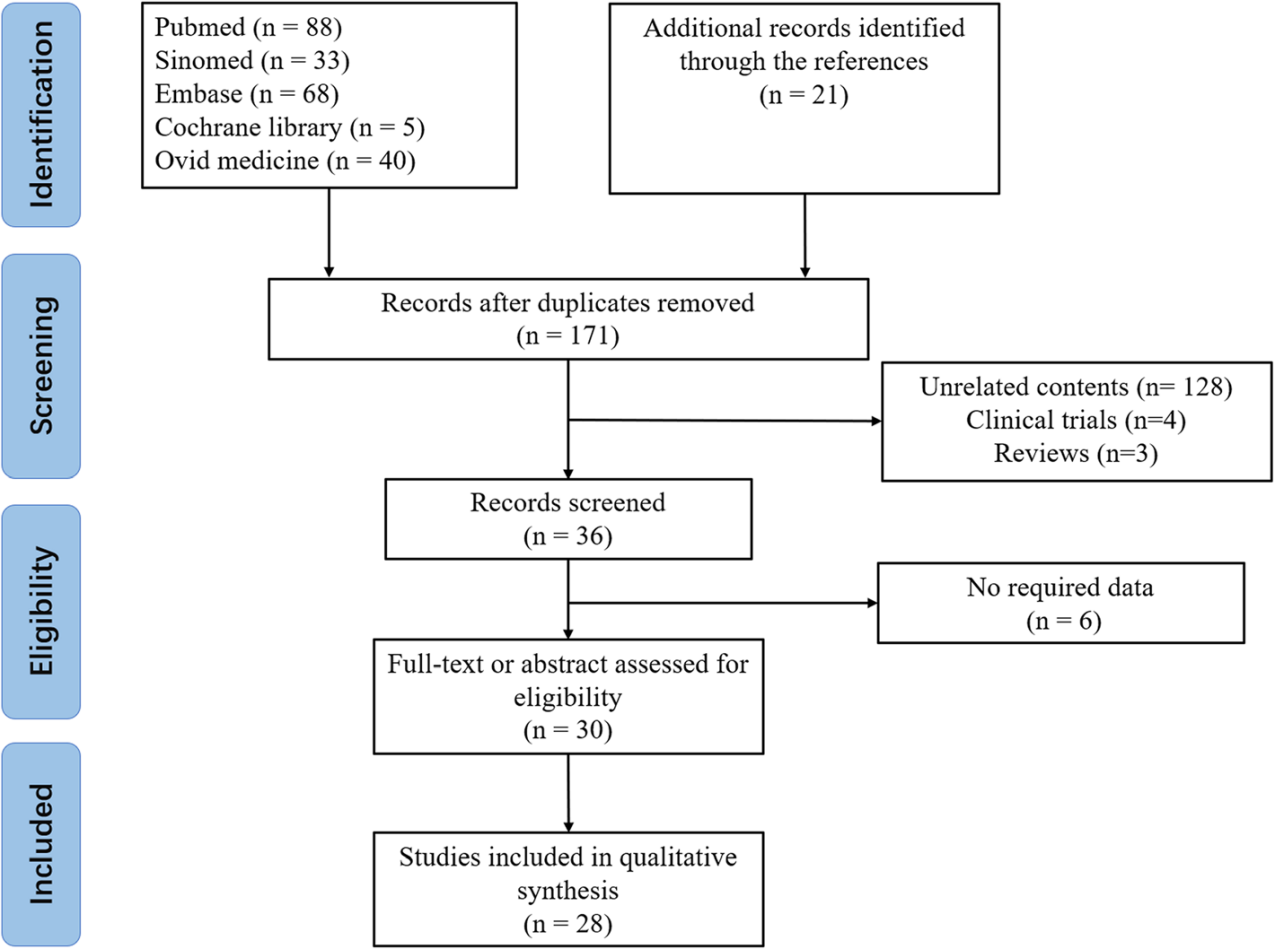
Trial inclusion and exclusion flow chart. Using (Cystic Fibrosis OR CFTR) AND (Chinese OR China) as the key words, limited to title or abstract, 255 records were retrieved from PubMed, Cochrane library, Embase, OVID medicine and SinoMed. A total of 84 duplicate articles, 135 unrelated articles, 6 articles without required data and 2 articles reporting duplicated cases were excluded

The process of study searching and including was shown in Fig. 1. In total, 71 Chinese patients described in 28 references were included in this article. Among them, 58 of them were from mainland China, 9 from Taiwan, and one from each of Australia, Canada, America and Singapore. [14–41]

### Clinical manifestations of Chinese CF patients

Within the 71 patients, 17 had a definite or suspected family history of CF and one patient was from a consanguineous family. The symptom onset age ranges from newborn to 14 years old (median age: 1 y; interquartile range (IQR): 0.1–6.0 y), while the age at diagnosis ranges from four months to 28 years old (median age: 10 y; IQR: 4.6–13.3 y). Certain details were not available in the case of two siblings from Taiwan. [27]

The respiratory tract is the most commonly affected system in Chinese patients. Sinus and pulmonary diseases including sinusitis (33/71, 46.5%) and bronchiectasis (67/71, 94.4%) are most commonly seen. Fifteen of the 71 (15/71, 21.1%) Chinese patients exhibited ABPA. Out of 38 patients who underwent pulmonary function testing, 31 (31/38, 81.6%) had a FEV_1_ below 80% predicted. *Pseudomonas aeruginosa* (*PA*) is the most commonly seen pathogen in Chinese CF patients with a ratio of 46/59 (78.0%).

Forty-two (42/71, 59.2%) patients had digestive system symptoms including chronic diarrhea, steatorrhea, ileus, hepatomegaly, hepatocirrhosis, splenomegaly or pancreatic insufficiency (PI). Ten (10/71, 14.1%) Chinese patients exhibited PI.

Among the 31 male patients, only one exceeded 18 years of age. The patient accepted urography and semen tests and was diagnosed with CBAVD.

Sweat chloride testing results were obtained for 52 patients and all of them had elevated sweat chloride concentrations with a median level of 118.0 mmol/L (IQR: 104.0–135.0 mmol/L). Fifteen patients had died at the time of report with the age of death ranging from 0.33 y to 24 y. All of these patients died of respiratory failure or severe pulmonary infection. Detailed information of clinical features of the 71 Chinese CF patients was summarized in Table 1.

**Table 1:** Clinical manifestations of Chinese CF patients

CF	*N* = 71		
Age at onset(y)(*n* = 63)	Median age 1y	Pulmonary function	
	0.1–6.0y*	FEV_1_(*n* = 38)	67.8% ± 20.9%
Age at diagnosis(y)(*n* = 69)	Median age 10y	FVC(*n* = 20)	80.7% ± 13.8%
	4.6–13.3y^*^	FEV_1_/FVC (*n* = 19)	71.2% ± 12.3%
Sex(F/M) (*n* = 69)	37/32	Sweat chloride test (*n* = 52)	Median value 118.0 mmol/L
Family history(*n* = 71)	17/71		104.0–135.0 mol/L^*^
Clinical manifestations		Sputum or BALF culture	
Underweight (*n* = 25)		(*n* = 59)	
normal	8/25	*PA*	46/59
-1 SD - 0	2/25	*MSSA*	13/59
-2 SD - (−1 SD)	6/25	*Aspergillus*	4/59
−3 SD - (− 2 SD)	3/25	*KP*	3/59
<-3 SD	6/25	Other	3/59
Respiratory system (n = 71)		*MRSA*	2/59
bronchiectasis	67/71	*E.Coli*	1/59
ABPA	15/71	*MRSE*	1/59
sinusitis	33/71	*MSSE*	1/59
Digestive system (*n* = 71)	*n* = 71	*SP*	1/59
icterus	0	*BC*	1/59
diarrhea	15/71	*HI*	1/59
hepatomegaly	1/71	*AC*	1/59
hypersplenotrophy	1/71	*MC*	1/59
ileus	3/71	NTM	1/59
hepatolienomegaly	2/71	Misdiagnosis (*n* = 29)	
positive fecal Sudan III	10/71	bronchiectasis	12/29
hepatocirrhosis	2/71	pneumonia	9/29
Reproductive system (*n* = 38)		Batter syndrome	5/29
		ABPA	5/29
CBAVD (adult male, *n* = 1)	1	other	3/29
		TB	4/29
		upper respiratory tract infection	2/29

*:IQR

*ABPA* allergic bronchopulmonary aspergillosis, *Ac Acinetobacter*. *BALF* bronchial alveolar lavage fluid, *BC Burkholderiacepaci, CBAVD* congenital bilateral absence of the vas deferens, *CF* cystic fibrosis, *E.coli Escherichia coli*, *FEV*_*1*_ forced expiratory volume in the first second, *FVC* forced vital capacity, *HI Haemophilus influenza*, *KP Klebsiella pneumoniae*, *MC Moraxella catarrhalis*, *MRSA* methicillin-resistant *Staphylococcus aureus*, *MRSE* methicillin-resistant *Staphylococcus epidermidis*, *MSSA* methicillin-sensitive *Staphylococcus aureus*, *MSSE* methicillin-sensitive *Staphylococcus epidermidi, NTM* non-tuberculosis *Mycobacterium*, *PA Pseudomonas aeruginosa*, *PI* pancreatic insufficiency, *SD* standard deviation, *SP Streptococcus pneumoniae*. *TB* tuberculous bacillus

### Distinct *CFTR* variant spectrum among Chinese CF patients

Since the first case of CF in Mainland China identified by DNA analysis in 1995, [20] 61 Chinese CF patients, including patients from Mainland China, Taiwan and other counties of Chinese origin, have been diagnosed with definite *CFTR* variants. There were, in total, 59 different *CFTR* variants identified on over 120 Chinese chromosomes. Mutation p.Gly970Asp (c.2909G > A, p.G970D) was found to be the most frequent variant among Chinese CF patients with an allele frequency of 9.8% (12/122). Meanwhile, the second and third most common variants were c.1766 + 5G > T (legacy name 1898 + 5G- > T; 7.4% [9/122]) and p.Ile1023Arg (c.3068 T > G, p.I1023R; 4.9% [6/122]), respectively. The majority of *CFTR* variants in Chinese have been observed only once and are very rare or absent in Caucasians. Variants observed more than once in Chinese CF patients are listed in Table 2.

**Table 2:** Frequency of *CFTR* variants among Chinese CF patients

Nucleotide change	Amino acid change	Frequency	Percent (%)
c.2909G > A	p.G970D	12	9.8
c.1766 + 5G > T	–	9	7.4
c.3068 T > G	p.I1023R	6	4.9
c.263 T > G	p.L88X	5	4.1
c.293A > G	p.Q98R	5	4.1
c.1666A > G	p.I556V	4	3.3
c.595C > T	p.H199Y	4	3.3
c.1657C > T	p.R553X	3	2.5
c.3196C > T	p.R1066C	3	2.5
c.326A > G	p.Y109C	3	2.5
c.648G > A	p.W216X	3	2.5
△E20 (c.3140-454_c.3367 + 259del931ins13)	p.R1048_G1123del	2	1.6
c.1000C > T	p.R334W	2	1.6
c.[2083dupG; 2684G > A]	p.[E695Gfs*35; S895 N]	2	1.6
c.2374C > T	p.R792X	2	1.6
c.414_415insCTA	p.L138_H139insL	2	1.6
c.558C > G	p.N186K	2	1.6

## Discussions

CF is one of the most common life-threatening autosomal recessive diseases in Caucasians, among which clinical features and the *CFTR* mutation spectrum are well defined. However, there is a significant under-recognition of CF by Chinese physicians. The median age at diagnosis of CF in Chinese patients is 10 years, compared with 0.5 months in Europe [42] and 3 months in America reported in the 2017 Patient Registry Annual Data Report (available at https://www.cff.org/Research/Researcher-Resources/Patient-Registry/), suggesting a significant delay in diagnosis among the Chinese population. Even for the 13 patients from outside of Mainland China (including Taiwan and other countries), the median age at diagnosis is 4 years. Though better than Mainland China, this is still significantly delayed when compared with the Western world.

Typical CF symptoms often affect multiple systems, especially involving the lungs, pancreas, digestive tract and reproductive system in males. Nevertheless, those typical CF-like symptoms do not usually happen concurrently in CF individuals of Chinese origin. Therefore, Chinese physicians often make wrong diagnoses such as tuberculosis, bronchiectasis, pulmonary infection and diarrhea, which are widespread CF-like diseases in Asia. This under-recognition, has greatly contributed to the diagnostic delay of CF among Chinese patients.

The most commonly affected system in Chinese CF patients is the respiratory system. Chronic airway infections, mucus in airways, inflammation, progressive bronchiectasis, and decline of pulmonary function are the main reasons for the death of CF patients in Chinese, which is similar to the West. Interestingly, the rate of ABPA in Chinese patients reached a slightly higher rate than the 5.1% reported in American patients (https://www.cff.org/Research/Researcher-Resources/Patient-Registry/). This may be due to selection bias as some patients visited a doctor specifically for ABPA in China.

**Fig. 2:**
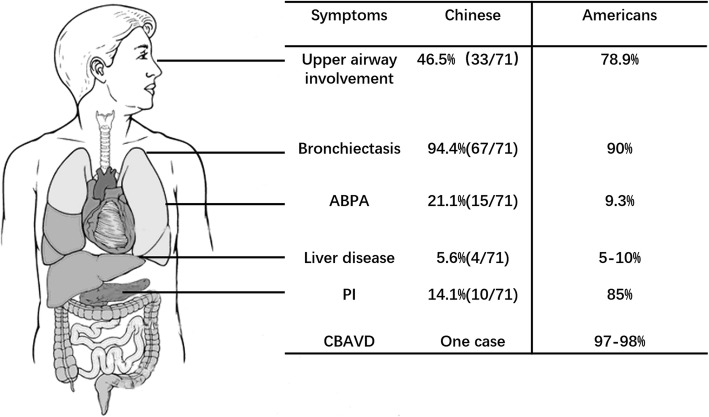
Comparison of clinical features between Chinese and Caucasian CF patients. The clinical statistics of Caucasians were collected according to the 2017 Cystic Fibrosis Foundation Patient Registry Annual Data Report (available at https://www.cff.org/Research/Researcher-Resources/Patient-Registry/). *ABPA* Allergic bronchopulmonary aspergillosis, *PI* Pancreatic insufficiency, *CBAVD* Congenital bilateral absence of the vas deferens

Apart from the respiratory findings, symptoms affecting other organs in Chinese CF patients are not as commonly seen as in Caucasians. One obvious example is the much lower frequency of PI (14.1%) among Chinese patients compared with the frequency of about 85% in Caucasians. (https://www.cff.org/Research/Researcher-Resources/Patient-Registry/). The inaccessibility of PI testing technology in China may partially explain this discrepancy. Furthermore, since p.Phe508del (c.1521_1523delCTT, p.F508del) is the most common *CFTR* gene mutation in Caucasians, which was reported to be associated with PI. Low incidence of PI in Chinese CF patients might be attributed to the marked genetic heterogeneity compared to Caucasians (see below). Male infertility owing to CBAVD is commonly seen in Caucasian CF patients. [43] However, we cannot draw the same conclusion for Chinese patients because there was only one adult male CF patient reported in the Chinese population. Further studies with a longer follow-up time and a larger sample size are required to answer this question. Differences of the clinical manifestations of CF between Chinese and Caucasian patients are summarized in Fig. 2.

As one of the criteria, sweat chloride testing was conducted in 73.2% (52/71) patients in this cohort. Not all patients had access to the test because it is only available in very limited number of large medical centers in China. As a non-invasive diagnostic method, its promotion and popularization will contribute to a decrease in the under-diagnosis of CF in China. Furthermore, Chinese CF patients are more likely to have negative family history, possibly caused by the one-child policy in China. With the recent implementation of the two-child policy, there may be more CF patients with family history observed, which may make the diagnosis of CF less hidden.

From the clinical manifestations in Chinese CF patients mentioned above, we summarized the situations when physicians should keep these diagnostic clues in mind for Chinese CF patients in Table 3.

**Table 3:** Patients suggested to have diagnostic tests of CF

1. Young sporadic bronchiectasis patient	
2. Bronchiectasis patients with upper lobes predominately affected	
3. Young bronchiectasis patients that are PA positive in sputum	
4. Young bronchiectasis patients with rhinitis or nasal polyp	
5. Young bronchiectasis patients with digestive tract symptoms	
6. Bronchiectasis patients with positive family history	
7. Bronchiectasis patients with infertility	

**Fig. 3:**
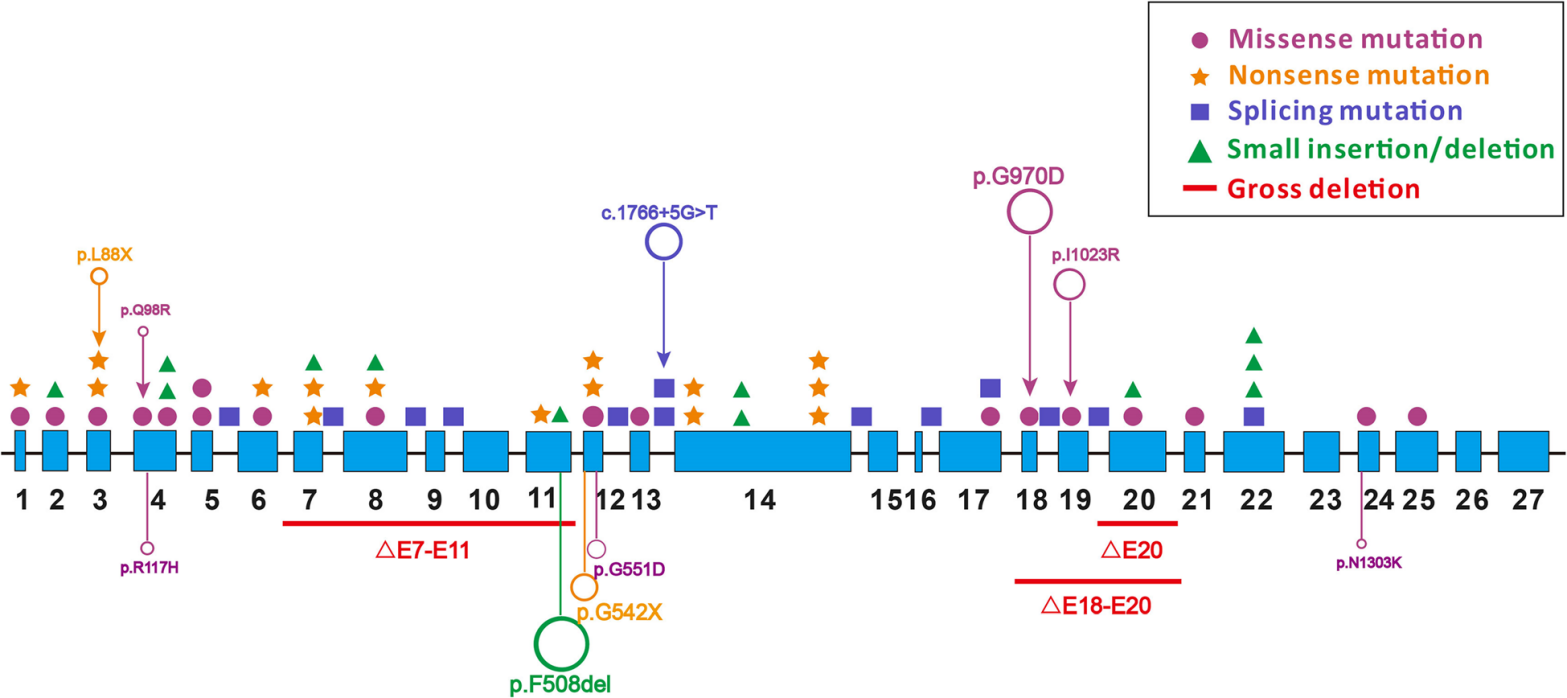
Comparison of *CFTR* variant spectrums between Chinese and Caucasian CF patients. Mutations of different types were presented in different colors and shapes. Open circles indicated the 5 most common mutations found in Chinese (upper panel) OR Caucasian (lower panel) patients with CF. The size of each circle represents its relative frequency in corresponding race, but not drawn totally to scale. No overlap of the most common mutations was observed between two populations

Over the past three decades, the variant spectrum of *CFTR* among Caucasians in Western countries has been well established. Meanwhile, our data shows minimal overlap between the variant spectrums of Chinese and Caucasian CF patients (Fig. 3). For example, the most frequent mutation in Caucasians, p.F508del, has been observed only once in Chinese population, contrasting with a frequency of about 70% in the West. [44] In the CF population carrier screening panel recommended by the American College of Medical Genetics (ACMG), the 23 most common *CFTR* variants cover about 84% of CF-causing mutations among Caucasians. [45] By comparison, only four of these mutations (p.Arg334Trp, [34]p.Arg553X, [32]p.F508del, [35] and p.Asn1303Lys [34]) have been observed in Chinese CF patients, accounting for no more than 6% Chinese CF alleles. Furthermore, the three most common variants (c.2909G > A, p.G970D; c.1766 + 5G > T; c.3068 T > G, p.I1023R) identified in Chinese are all restricted to patients of Chinese origin. Besides these three, the majority of *CFTR* variants in Chinese is very rare or absent in Caucasians. Differences in genetics have contributed to the low diagnostic rate in China, as well as in other races of the world. A distinct *CFTR* mutation spectrum might lead to a considerably different phenotype, evidenced by the atypical manifestations in Chinese CF patients. In addition, during *CFTR* mutation screening among Chinese patients, use of the Caucasian panel will certainly result in false negative results. That is, a Chinese-specific CF panel is warranted for *CFTR* variant screening among Chinese CF patients as well as among Chinese immigrants in other countries.

Correct functional characterization of each mutation is vital to realize the goal of personalized medicine. Great effort has been made in functional testing of *CFTR* mutations found in Caucasians by groups like CFTR2 team [46] and CFTR-France. [47] However, as previously mentioned, most CF-causing alleles found in Chinese patients are very rare or absent in other races and have not yet been analyzed for their functional consequences. This restricts Chinese CF individuals from benefiting from the most up-to-date advancements about CF treatment in the West. A good example is the promising small molecule therapeutic drugs, which work in patients with mutations of specific classes. [48, 49]

Interestingly, in the Chinese *CFTR* variant spectrum, though not yet well-established, only four CF alleles with gross rearrangement events were observed, including one allele with △E7-E11 (c.744-?_1584 +?del), [32] one with △E18-E20 (c.2909-?_3367 +?del), [32] and two with △E20 (c.3140-454_c.3367 + 249del931ins13). [39] It is worth mentioning that the four gross deletions were all identified in a single center from Beijing, China. According to the total number of CF patients enrolled in their lab, despite the relatively small sample size and possible selection bias, the allele frequency of gross rearrangements accounted for 12.5% (4/32) of all CF-causing alleles in Chinese patients. However, no gross rearrangement event has ever been observed in CF patients of Chinese origin in any other lab, although there have been over 60 patients and 120 chromosomes reported in the Chinese population. Overlook of potential gross genomic rearrangements and the inaccessibility of multiplex ligation-dependent probe amplification (MLPA) analysis in many labs is likely responsible for this possibly underestimated rearrangement rate, which has in turn contributed to the under-recognition of CF. Therefore, further emphasis should be placed on the necessity of MLPA analysis in routine *CFTR* mutation screening in Chinese patients, especially for those with only one or no mutations identified via exon sequencing. In addition, the screening data provided by WuXi NextCODE Ltd. was based on next-generation sequencing, without MLPA analysis of the *CFTR* gene. Thus, the aforementioned carrier frequency of 1/127 might be also underestimated.

## Perspective

CF involves multiple organs. As such, successful treatment requires multidisciplinary cooperation. However, CF patients in China are currently severely under-recognized with a high rate of misdiagnosis and missed diagnosis. This, in conjunction with the huge cost of lung transplant and limited source of donors, makes CF difficult to treat in China. Currently, treatment strategies for CF in China mainly focus on preventing infection and providing nutritional support. Out of the 71 CF patients reviewed in this manuscript, 15 had passed away at the time of publication. The age of death ranges from 0.33 y to 24 y based on available data, not even close to the life expectation of 43.6 y for individuals born from 2013 to 2017 in the US, recorded in the 2017 Patient Registry Annual Data Report (available at https://www.cff.org/Research/Researcher-Resources/Patient-Registry/). Therefore, with reasonable treatment, Chinese CF patients should achieve an extended life expectancy.

Recently, small molecular drugs have shown great potential in treating CF. CFTR correctors (e.g. lumacaftor, VX-809) [47], potentiators (e.g. ivacaftor, VX-770) [49] and the combination therapy [50] have provided patients with specific mutations great progress on their disease control. Unfortunately, there are few studies on the molecular consequences of Chinese-specific *CFTR* variants. The most frequent *CFTR* mutation in Chinese, p.G970D, is predicted to be a gating mutation which makes an extensive in vitro functional characterization feasible. Meanwhile, Chinese CF patients will benefit from the recently launched National Rare Diseases Registration System of China (www.nrdrs.org.cn) and multidisciplinary cooperation. Moreover, the setting up of this registration system could be the beginning of a better reporting of CF in China, which will help to improve data-input, consistency in definitions and, more importantly, the recognition of CF by Chinese physicians.

## Strengths and weaknesses

This paper summarized the cases of CF previously reported in Chinese populations, compared the clinical characteristics of Chinese and Caucasian CF patients for the first time, and emphasized the significant differences in the *CFTR* mutation spectrum in Chinese when compared to Caucasians. However, data on the relative rates of occurrence of various characteristics of CF patients in China are uncertain due to small numbers of patients and varying degrees of ascertainment bias. The article is a systemic review of the reported cases and patients, spanning almost 40 years, with some cases being lost to follow up.

## Conclusion

Due to the atypical clinical symptoms, marked genetic heterogeneity, the inaccessibility of sweat and genetic testing facilities and the one-child policy in China, CF is significantly under recognized in China. Emerging evidence has indicated a potential larger number of Chinese CF patients. Therefore, much more attention should be put on studies about the phenotype and genotype of CF patients not only for Chinese physicians and researchers, but also for those working in ethnic Chinese-intensive areas. To benefit Chinese-origin CF patients, more effective and precise *CFTR* screening platforms are urgently needed. Another great challenge is to characterize the consequences of *CFTR* mutations in Chinese, which will help to realize personalized treatment. Fortunately, a number of studies are in progress as the national registration of rare diseases in China has recently been launched which will bring a brighter outlook for Chinese CF patients, even extending to other Asian countries.

## Abbreviations

ABPA: Allergic bronchopulmonary aspergillosis
*Ac*: *Acinetobacter*
BALF: Bronchial alveolar lavage fluid
*BC*: *Burkholderiacepacia*
CBAVD: Congenital bilateral absence of the vas deferens
CF: Cystic fibrosis
CFTR: Cystic fibrosis transmembrane conductance regulator
DNA: Deoxyribonucleic acid
*E.coli*: *Escherichia coli*
FEV_1_: Forced expiratory volume in the first second
FVC: Forced vital capacity
*HI*: *Haemophilus influenza*
IQR: Interquartile range
*KP*: *Klebsiella pneumoniae*
*MC*: *Moraxella catarrhalis*
MLPA: Multiplex ligation-dependent probe amplification
MRSA: Methicillin-resistant *Staphylococcus aureus*
MRSE: Methicillin-resistant *Staphylococcus epidermidis*
MSSA: Methicillin-sensitive *Staphylococcus aureus*
MSSE: Methicillin-sensitive *Staphylococcus epidermidis*
NBS: Newborn screenings
NTM: Non-tuberculosis *Mycobacterium*
*PA*: *Pseudomonas aeruginosa*
PI: Pancreatic insufficiency
SD: Standard deviation
*SP*: *Streptococcus pneumoniae*
TB: Tuberculous bacillus
